# Acute Pancreatitis: A Possible Side Effect of COVID-19 Vaccine

**DOI:** 10.7759/cureus.14741

**Published:** 2021-04-28

**Authors:** Om Parkash, Artem Sharko, Aneeba Farooqi, Grace W Ying, Prashant Sura

**Affiliations:** 1 Internal Medicine, Chicago Medical School, Rosalind Franklin University of Medicine and Science, North Chicago, USA; 2 Internal Medicine, Rosalind Franklin University of Medicine and Science, Northwestern Medicine McHenry Hospital, McHenry, USA

**Keywords:** covid-19, acute pancreatitis, pfizer-biontech, bnt162b2 mrna

## Abstract

For the first time, the mRNA technology was utilized to produce a vaccine against COVID-19 after the unprecedented pandemic equally affected every part of the world. Pfizer-BioNTech (BNT162b2) mRNA vaccine against severe acute respiratory syndrome coronavirus 2 (SARS-CoV-2) was granted emergency use authorization (EUA) by Food and Drug Administration (FDA) in December 2020. EUA has been widely discussed in the medical literature and the general public. The safety of the BNT162b2 vaccine has been investigated in short-term trials with data available for three months. We present a case of a 96-year-old female with a past surgical history of cholecystectomy who presented with acute onset severe abdominal pain a few days after getting the first dose of Pfizer-BioNTech COVID-19 vaccine. She was diagnosed with acute pancreatitis with a lipase level of 4036 U/L. Extensive history and investigations were unable to find any etiology. The patient was conservatively managed and discharged home without any complications. There has been some data available in medical literature showing an association between acute pancreatitis and COVID-19 infection. Trial data of Pfizer COVID-19 also shows one case of acute pancreatitis in the treatment group. There have also been individual cases of unexplained acute pancreatitis shared by medical professionals on online forums. Our main goal to write this case is to make medical literature aware of possible emerging side effects of the COVID-19 vaccine, one of such side effects being self-resolving uncomplicated acute pancreatitis.

## Introduction

For the first time, the mRNA technology was utilized to produce a vaccine against COVID-19 after the unprecedented pandemic equally affected every part of the world. Pfizer-BioNTech (BNT162b2) mRNA vaccine against severe acute respiratory syndrome coronavirus 2 (SARS-CoV-2) was granted emergency use authorization (EUA) by Food and Drug Administration (FDA) in December 2020. EUA has been widely discussed in the medical fraternity and the general public. Long-term side effects of mRNA vaccines could not be established at the time of EUA due to the limited availability of trial data [1,2]. FDA has been continuously monitoring the post-marketing long-term or rare side effects. By reporting this case, our goal is to highlight and make the medical literature aware of the possible effects of this BNT162b2 mRNA vaccine. We also want to emphasize the role of healthcare workers in exploring the emerging long-term side effects of this new product. This information may be essential to make timely interventions and create guidelines to protect high-risk individuals.

## Case presentation

The patient is a 96-year-old Caucasian female who presented to the emergency department with complaints of progressively worsening epigastric pain for two days. The pain was sharp, 10/10 in intensity, and radiating to the right lower chest. She had some nausea but no vomiting, diarrhea, or fever. The patient also denied any recent sick contacts, diet changes, or use of new medical preparations or herbal products. Her past medical history includes diastolic congestive heart failure, hypertension, and hypothyroidism. Past surgical history was significant for cholecystectomy and appendectomy. Her home medications included amlodipine, aspirin, bumetanide, carvedilol, levothyroxine, and lisinopril, which has been her regimen for the past three years without any recent changes. The patient denied any use of alcohol in the last 10 years. Notably, the patient got the first dose of the BNT162b2 COVID-19 vaccine a few days before coming to the hospital.

On physical examination, the patient had epigastric abdominal tenderness, but otherwise, the exam and vital signs were within normal limits. Initial lab work including complete blood count (CBC), comprehensive metabolic panel (CMP), urinalysis, lipid panel, thyroid-stimulating hormone (TSH), troponin levels, and electrocardiogram (EKG) were unremarkable. Her lipase level was significantly elevated at 4036 U/L. Computed tomography (CT) scan of the abdomen did not show any acute pathological findings. The patient was diagnosed with acute pancreatitis and monitored overnight with conservative treatment. Remarkably, her pain was already improving on admission and nearly resolved overnight. Lipase levels trended down to 205 U/L. Her diet was gradually advanced to regular. The patient's symptoms resolved, and she was discharged home in stable condition. The brief duration of symptoms and the timing of COVID-19 vaccination administration led to the increased likelihood of the vaccine as a plausible culprit for her acute bout of pancreatitis.

## Discussion

Acute pancreatitis, defined as inflammation of the pancreas leading to autodigestive injury to the pancreatic parenchyma, can develop due to multiple causes. Some of the most common factors include gallstones, hypertriglyceridemia, alcohol, and smoking. Toxins or drugs, autoimmune, idiopathic, and procedure-related infections are few other less frequent causes [3].Autoimmune pancreatitis is usually diagnosed at a much younger age and, in most cases, associated with other autoimmune conditions [4]. In the patient mentioned in this article, all common risk factors were ruled out. Multiple cases of COVID-19-induced pancreatitis have been reported in the literature [5-7]. Medications have also been linked to acute pancreatitis. A case of lisinopril-induced pancreatitis has been reported in the past after starting on lisinopril two weeks before presentation [8]. It is unlikely that this could be the cause in our patient as she had been on the medication for many years before developing the episode.

Since the beginning of the COVID-19 pandemic, the greatest hope has been developing a vaccine effective against SARS-CoV-2. Now we have several options available, which have proven to be highly effective and safe enough to be used under EUA. Long-term side effects were not established at the time of EUA due to the unavailability of long-term data. Only three months of trial data were used for EUA. It is, however, essential to remember the potential side effects that these vaccines may have. One of these side effects can be acute pancreatitis. The safety of the BNT162b2 vaccine has been investigated in short-term trials with data available for three months, and local reactions were observed predominantly in patients who received the vaccine when compared to the placebo group [1,2]. The most common local reaction was pain at the injection site, with 66%-83% of recipients, depending on the age, reporting the reaction and only 8%-14% of placebo recipients developing the symptom [1,2]. Systemic reactions such as headache, fatigue, and fever were also observed, predominantly in the younger population, with headache and fatigue being reported by more than 52%-59% of vaccine recipients, and fever was reported by 11%-16% of recipients [1]. Some of the recipients also reported severe adverse events between the first dose and the second dose that was given after one month. One of them developed pancreatitis, and none of the placebo recipients had such an event [2]. 

Although it is difficult to make conclusions about the likelihood of the vaccine being the etiologic factor of pancreatitis, it is essential to continue monitoring for possible under-reported side effects until we have extensive long-term data available in post-marketing surveillance for long-term and rare side effects. Because the vaccine is new, it is reasonable to assume that we have not yet seen many of the potential side effects that may occur in patients who have received it. Our main goal to write this case is to make medical literature aware of the possible emerging side effects of the COVID-19 vaccine, one of such side effects self-resolving uncomplicated acute pancreatitis.

## Conclusions

Our patient developed acute pancreatitis several days after receiving the BNT162b2 vaccine and had no other risk factors for the condition. We present this case to make medical literature aware of the possibility that the vaccine may have some side effects that are not yet reported or taken into consideration. The incredible benefits of the vaccine are apparent and not disputed in this article. However, some patients may likely be at risk of developing some unestablished and high-risk side effects, and being aware of these risks will aid in weighing the risks versus benefits when deciding who should be vaccinated.
